# Research on thermal control technology for ultra-high-speed inter-satellite laser communication terminal

**DOI:** 10.1038/s41598-026-50135-8

**Published:** 2026-04-28

**Authors:** Shijun Li, Yanlong Han, Teng Xie, Shaojun Wu

**Affiliations:** Beijing Laser Starcom Technology Co., Ltd, Beijing, 100094 China

**Keywords:** Laser communication, Thermal design, Thermal simulation, Thermal balance test, New process, Engineering, Mathematics and computing, Optics and photonics

## Abstract

This paper focuses on the thermal control technology of a new generation ultra-high-speed inter-satellite laser communication terminal. The structure of the laser communication terminal, the influence of the satellite platform’s thermal interface, the difficulties in thermal design, and the thermal design schemes are explained in this paper. Thermal simulation and thermal balance tests are also conducted. In the thermal simulation, the temperature of the main optical system is 18.7℃~22.0℃, and in the thermal balance test, the temperature of the main optical system is 17.6℃ ~ 21.1℃, meeting the requirement of 20 ± 3℃. In addition, the reasons for the low temperature of the shafting components during the thermal test and the problems caused by the new wiring process were discussed, and solutions were given to provide a basis for the thermal design optimization of subsequent project batch production.

## Introduction

In recent years, the construction of global satellite constellations has entered a period of rapid development. With the continuous expansion of the scale of large-scale low-orbit satellite constellations and the sharp increase in data transmission demands, inter-satellite laser communication technology is gradually becoming one of the means to replace traditional microwave communication technology, with its advantages of high bandwidth, low latency, strong anti-interference ability, and high security^1^. However, inter-satellite laser communication system faces complex and changeable thermal environments during orbit operation, which poses severe challenges to the reliability and stability of the system.

As an important part of the inter-satellite laser communication system, the main task of thermal control technology is to maintain the system working within a suitable temperature range and ensure the stability of optical components and communication quality. In the space environment, the system not only has to endure extreme cold and dark conditions, but also needs to withstand the impacts of complex external heat flux such as solar radiation, Earth’s infrared radiation, and Earth’s reflection. Therefore, the development of efficient and reliable thermal control technology is crucial to inter-satellite laser communication systems. The basic principle of space thermal control technology is to achieve effective management and control of system heat, with reasonable thermal design and the heat transfer methods of conduction and radiation.

Since the 21st century, countries such as Europe, the United States, and China have developed multiple laser communication terminals and conducted in-orbit technical verification, which has greatly promoted the development and application of laser communication technology. Among the notable projects are LLCD^2^, TBIRD^3^, LCTSX^4^ and “Micius“^5^. With the rapid development of laser communication technology, researchers have conducted extensive studies on thermal control technology and thermal deformation effects in inter-satellite laser communication terminals, mainly including thermal design of high-precision 2D gimbal servo mechanism^6^, thermal control technology for large-aperture optical antenna^7^, thermal control technology for high-power optical device^8^ high-precision temperature control technology^9^, thermal design and thermal test verification of the entire system^10^, and the influence of thermal deformation of optical antenna and reflector^11–14^. However, in the previous thermal design process of laser communication terminals, researchers paid more attention to the thermal design research of components or entire system. Few people mentioned the impact of the satellite platform installation interface on the heat transfer and radiation of the terminal. Moreover, there is currently a lack of special research on thermal design of ultra-high-speed inter-satellite laser communication terminal.

The research subject of this article is a newly-developed ultra-high-speed inter-satellite laser communication terminal. This terminal is modular, flexible and scalable, and also has a high transmission rate (≥ 100 Gbps). This paper takes it as the research object. On the basis of fully considering the space environment and structural characteristics, it integrates the thermal influence of the platform installation interface. Through the combination of active thermal control and passive thermal control, a detailed thermal design is carried out on the terminal. Moreover, thermal simulation and thermal balance test are carried out to ensure the reliability of the thermal control system.

## Introduction to laser communication terminal

### Structure

**Fig. 1:**
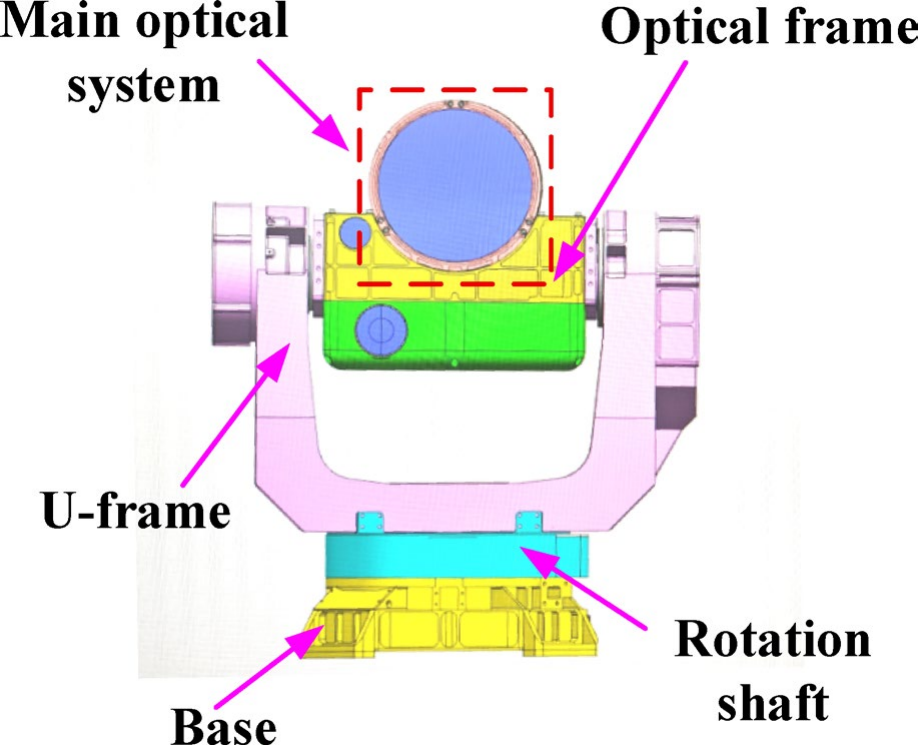
Schematic diagram of the structure of the laser communication terminal.

The structure of the new laser communication terminal is shown in Fig. 1, mainly including the main optical system (lens cone, primary mirror, secondary mirror), shaft components (base, rotation shaft, U-frame) and optical frame. The terminal’s total envelope size is 408 mm × 268 mm × 402 mm, and its weight is about 15 kg, with the characteristics of modularity, flexibility and scalability. The laser communication terminal has a large tracking range when working in-orbit, with the rotation range of the azimuth angle from − 90° to 90° and the pitch angle from − 60° to 85°.

### Mission

The laser communication terminal is installed on the satellite. The satellite operates in a circular orbit with a height of 500 km, and its orbital inclination is 55°. A three-axis stable attitude towards the ground is adopted by the satellite, with its forward flight direction defined as the + X direction and the direction towards the ground defined as the + Z direction. It is required that the maximum communication distance is greater than 4800 km and the maximum communication rate is 100 Gbps. The thermal control system maintains the temperature of its main optical system at 20 ± 3℃, the optical frame at 20 ± 5℃, and the shafting components at 20 ± 15℃.

## Analysis of thermal interface effects on satellite platform

The laser communication terminal is fixed on the installation surface of the satellite platform, with a 4 K cold black vacuum environment. The main ways of heat exchange with the outside world are heat radiation and heat conduction.

During the satellite’s operation in-orbit, the temperature of its platform varies significantly (-15 ~ 45℃). The large temperature fluctuations will have a considerable impact on the laser communication terminal, thereby affecting its communication quality. Since the laser communication terminal and the satellite platform cannot be installed in adiabatic manner, it is very necessary to study the heat transfer between them. In order to unload the temperature fluctuations of the platform, the laser communication terminal is installed insulated with the platform, and the bottom of the laser communication terminal is coated with multiple layers of thermal insulation material (MLI). Being covered with multiple layers, the thermal radiation between the laser communication terminal and the satellite platform can be negligible. Therefore, the main method of heat transfer between laser communication terminal and satellite platforms is heat conduction.

**Fig. 2:**
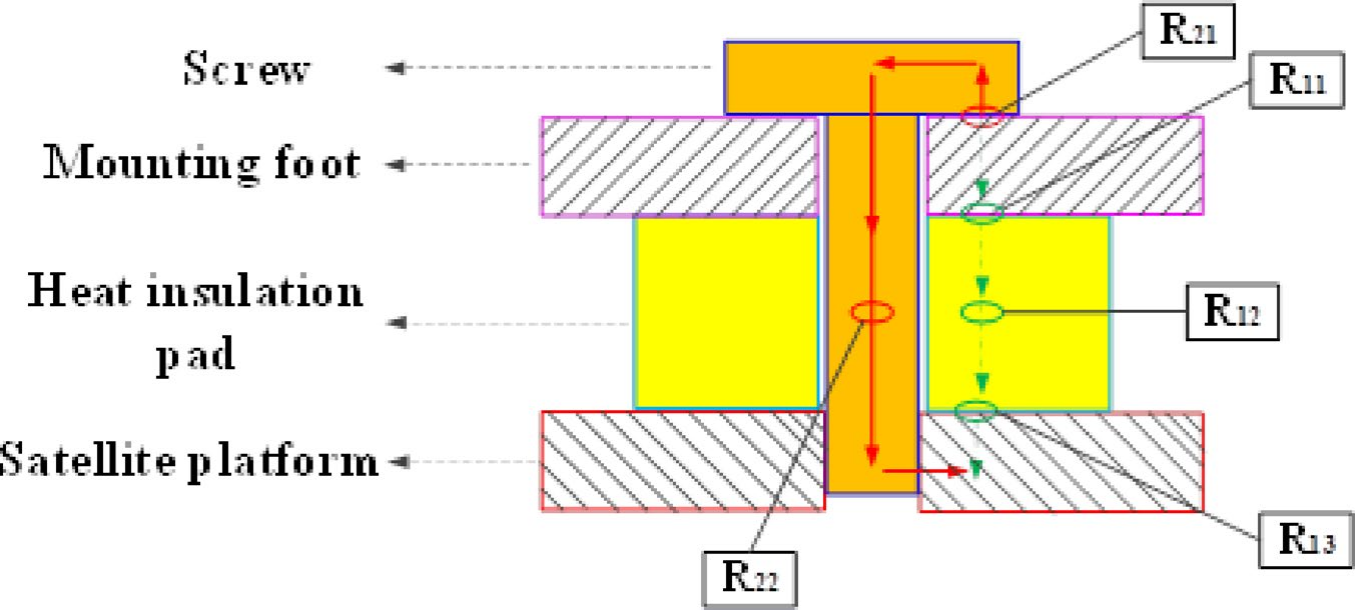
Schematic diagram of the connection between the satellite platform and laser communication terminal.

**Fig. 3:**
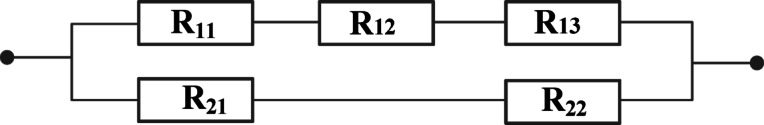
Schematic diagram of thermal resistance network between laser communication terminal and satellite platform.

The schematic diagram of the connection between the satellite platform and the laser communication terminal is shown in Fig. 2. The heat from the mounting foot of the laser communication terminal is transmitted to the satellite platform through two paths, which are in parallel. (1) Transmitted to the satellite platform via screws. (2) Transmitted to the satellite platform via heat insulation pad. The schematic diagram of the thermal resistance network between the laser communication terminal and the satellite platform is shown in Fig. 3, and the calculation relationships of each thermal resistance are presented in Table 1.

The calculation formula for the total thermal resistance (*R*) between the laser communication terminal and the satellite platform is as follows:

$$\:R=\frac{({R}_{11}+{R}_{12}+{R}_{13})\mathrm{*}({R}_{21}+{R}_{22})}{{R}_{11}+{R}_{12}+{R}_{13}+{R}_{21}+{R}_{22}}$$

**Table 1:** Calculation formulas for each thermal resistance.

Name	Definition	Formula
*R*_*11*_	Between the heat insulation pad and the mounting foot	$\:{R}_{i}=\frac{1}{{k}_{i}*{A}_{i}}$ *k*_*i*_ — Contact heat transfer coefficient (W/m^2^/K) $\:{A}_{i}$—Contact area (m^2^)
*R*_*13*_	Between the heat insulation pad and the platform	
*R*_*21*_	Between the mounting foot and screw	
*R*_*12*_	Conductive thermal resistance of the heat insulation pad	$\:{R}_{j}=\frac{{d}_{j}}{{\lambda\:}_{j}*{A}_{j}}$ *d*_*j*_ —Thickness (m) $\:{\lambda\:}_{j}$—Thermal conductivity (W/m/K) *A*_*j*_ —Cross-sectional area (m^2^)
*R*_*22*_	Conductive thermal resistance of the screw	

The insulation pad is made of fiberglass material(5 mm), and the screw is made of titanium alloy material, so the total thermal resistance of a single mounting foot is 35 K/W. Assuming that the temperature of the satellite platform is 40℃ and the mounting foot temperature is 15℃, the heat leakage (*Q*_*0*_) of a single mounting foot is:

$$\:{Q}_{0}=\frac{\varDelta\:T}{R}=\frac{40-15}{35}=0.71W$$

**Fig. 4:**
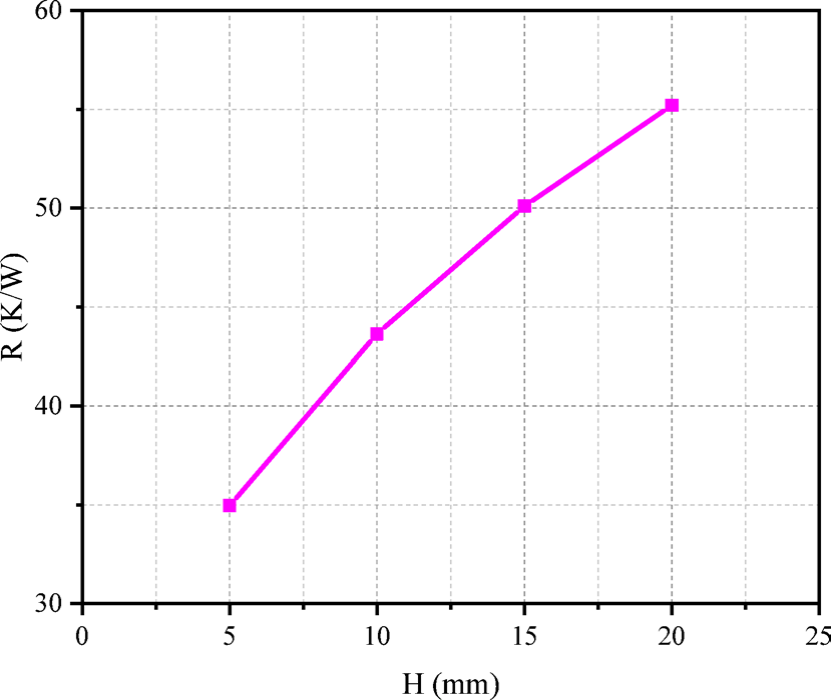
Relationship between H and R.

To study the relationship between the thickness of the heat insulation pad (*H*) and *R*, a corresponding relationship is plotted between them, as shown in Fig. 4, with other parameters remained unchanged.

As shown in Fig. 3, H is positively correlated with *R* (non-linearly), meaning that the thicker the insulation pad, the greater the total thermal resistance. Therefore, the insulation effect can be improved by increasing the thickness of the insulation pad.

## Thermal control system design

### Thermal control challenges

1. Compared with general loads, the temperature requirement of laser communication terminal optical system is very strict (20 ± 3℃).
2. The β-angle changes between − 78.4° and 78.4°, causing each surface to be exposed to sunlight alternately and making it difficult to select the radiating surface. Additionally, the external heat flux varies significantly, which can easily lead to temperature fluctuations.
3. The location of the laser communication terminal is severely blocked and it is difficult to dissipate heat.
4. The attitude of the laser communication terminal is highly complex and variable, bringing great challenges to thermal design and thermal simulation.
5. The beacon laser component has a small size and a large heating power density with per unit area during operation. Therefore, thermal design needs to give special attention to this part.
6. Large-scale of pitch and rotation poses significant difficulties to the layout of cables.

### Innovative solutions

The innovative solutions mainly lie in two aspects: (1) A structural and thermal integrated design is carried out for the optical frame, and aluminum-based silicon carbide material with high thermal conductivity and low linear expansion coefficient is selected. This not only improves the temperature uniformity of the optical frame but also reduces the impact of thermal deformation. Symmetrical radiating surfaces are opened on the outside of the optical frame to minimize the influence of non-symmetric external heat flux fluctuations. Additionally, an active heating zone is set up in the optical frame to create a 20 °C isothermal environment, meeting the high temperature requirements of optical components. (2) To address the issue of external cables affecting the two-dimensional rotation of the laser communication terminal, a new wiring process is adopted, hiding all cables inside the laser communication terminal.

### Thermal design

When designing the thermal control system, mature and flight-verified thermal control products and technologies are preferred to be selected. Furthermore, while ensuring reliability, safety and effectiveness, efforts should be made to minimize the cost of thermal control. In principle, except for the radiating surface, the outer surface of the laser communication terminal is covered with 15-unit MLI to isolate the influence of the space environment. Based on the difficulties in thermal control, this paper mainly focuses on the thermal design of optical frame, beacon laser component, as follows.

### Thermal design of optical frame

To reduce the mutual coupling effects among the components of the optical system, the optical components are designed in a modular way and integrated uniformly on the optical frame for heat dissipation. Meanwhile, the optical frame is built into an isothermal environment of around 20 °C through heating compensation, providing a stable installation environment for the optical components. The optical frame is made of aluminum-based silicon carbide material with high thermal conductivity and low linear expansion coefficient. Considering the alternating changes of external heat flux, the radiating surfaces are selected to be symmetrically arranged in the optical frame along the + X, -X, +Y and -Y directions, reducing the influence of each surface being exposed to sunlight alternately. The optical frame’s radiating surface is sprayed with KS-ZA white paint, covering an area of approximately 0.06 m².

**Fig. 5:**
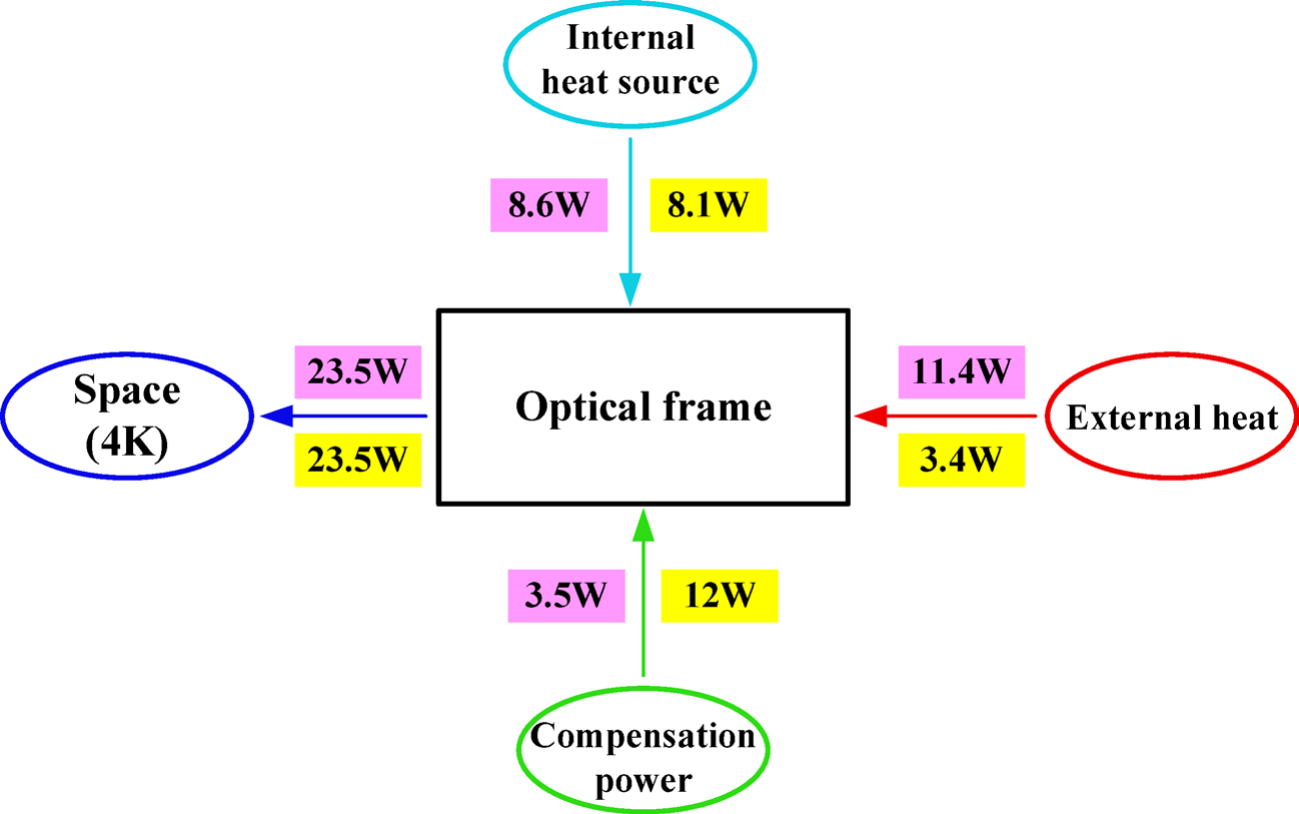
The schematic diagram of the energy flow of the optical frame.

Taking the temperature of the optical frame as 20℃ (*T*_*1*_ = 293.15 K), according to formula (3), it can be estimated that its maximum heat dissipation capacity is about 23.5 W. In the hot-case, the internal heat source on the optical frame is 8.6 W, and the absorbed external heat is 11.4 W. The radiating surface of the optical frame can meet the heat dissipation requirements (23.5 W > (8.6 W + 11.4 W)). In the cold-case, the internal heat source on the optical frame is 8.1 W, and the absorbed external heat is 3.4 W. Therefore, the compensation power of the optical frame is approximately 12 W. The schematic diagram of the energy flow of the optical frame is shown in Fig. 5.

### Thermal design of beacon laser component

**Fig. 6:**
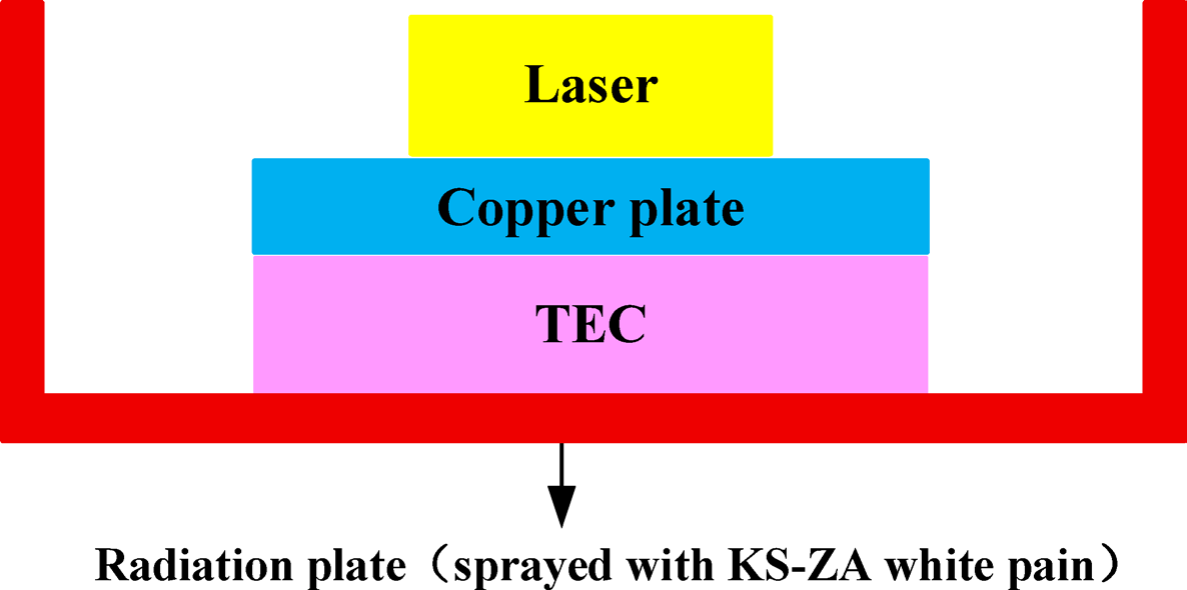
The schematic diagram of the thermal design of the beacon laser.

The size of the beacon laser is 16 mm×10 mm×6 mm, and the working duration of each orbital period is 5 min. the heat generation during working is 7 W, and the power density per unit area is 4.375 W/cm^2^, which brings great difficulties to thermal design. To solve the problem of high-power density per unit area of ​​beacon laser, the beacon laser is installed on a copper plate with a volume of 30 mm×30 mm×4 mm for heat expansion, reducing its power density per unit area. In addition, to ensure the stable operation of the beacon laser, a thermoelectric cooler (TEC) is used to refrigerate the copper plate, with the cold end temperature set at 41 °C. To satisfy the temperature difference requirements at the cold end and hot end of the TEC, the hot end of the TEC is thermally connected to the radiation plate for heat dissipation. The outer surface of the radiation plate is sprayed with KS-ZA white paint. Additionally, an active heating zone is set on the radiation plate to meet the temperature requirement when the laser is not working. The schematic diagram of the thermal design of the beacon laser is shown in Fig. 6.

### The design of the active heating circuit

In a low-temperature environment, it cannot meet the temperature needs of laser communication terminal, relying solely on thermal control technologies such as heat dissipation and insulation. At this time, heating technology is needed to ensure its normal operation or survival. The heating circuit, temperature sensor, and temperature control equipment are used together to form a closed-loop control circuit, providing precise temperature control for the laser communication terminal. A total of 10 active heating circuits are planned for the laser communication terminal as shown in Table 2, with a total heating power of 65 W. Each heating circuit is powered by a 12 V voltage.

**Table 2:** Statistics of heating circuits.

Number	Position	Design power(W)	Target temperature(℃)	Design resistance(Ω)
1	Lens cone	8	20	18
2	Secondary mirror	4	20	36
3	Primary mirror	5	20	28.8
4	1Optical frame1	12	20	12
5	2 Optical frame2	12	20	12
6	Beacon circuit box	6	10	24
7	1 U-frame 1	5	15	28.8
8	2 U-frame 2	5	15	28.8
9	Base	4	15	36
10	Rotation shaft	4	15	36

## Thermal analysis

### Theoretical foundation

#### The basic principle of thermal balance

When the spacecraft is in-orbit, its energy balance relationship is as shown in Eq. (1):

1 $${Q_1}+{Q_2}+{Q_3}+{Q_4}+{Q_5}={Q_6}+{Q_7}$$

*Q*_*1*_ represents the heat received by the spacecraft from solar radiation. *Q*_*2*_ represents the reflected heat from the Earth. *Q*_*3*_ represents the infrared radiation heat from the Earth. *Q*_*4*_ represents the background radiation heat in space (which can be ignored), *Q*_*5*_ represents the heat source inside the spacecraft, *Q*_*6*_ represents the radiation energy from the spacecraft to the universe, *Q*_*7*_ represents the change in internal energy of the spacecraft.

The laser communication terminal can be regarded as composed of many small-area units. Each unit can be considered as a node. The thermal balance equation for any node *i* is as follows:

2 $$\begin{gathered} \left( {{\alpha _{si}}S{\Phi _{1i}}+{\alpha _{si}}{E_r}{\Phi _{2i}}+{\varepsilon _{ei}}{E_e}{\Phi _{3i}}} \right){A_i}+\sum\limits_{{j=1}}^{N} {{B_{ji}}} {A_j}{\varepsilon _j}\sigma T_{j}^{4} \hfill \\ +{Q_i}+\sum\limits_{{j=1}}^{N} {{k_{ij}}} \left( {{T_j} - {T_i}} \right)={A_i}({\varepsilon _{ii}}+{\varepsilon _{ei}})\sigma T_{i}^{4}+{m_i}{c_i}\frac{{d{T_i}}}{{d\tau }} \hfill \\ \end{gathered}$$

here *A*_*i*_ is the area of the *i*_th_ surface, and *Φ*_*1i*_, *Φ*_*2i*_, and *Φ*_*3i*_, are the geometric angle coefficients of node *i* relative to the Sun, the Earth reflection and the infrared radiation, respectively. *S* is the solar constant (1322 ~ 1414 W/m^2^), *Er* is the intensity of the Earth’s reflection, and *E*_*e*_ is the intensity of the earth’s infrared radiation (237 W/m^2^). *α*_*si*_ is the solar absorptivity of node *i*, *ε*_*ei*_ is the infrared hemisphere emitter of node *i*, and *B*_*ji*_ is the radiation absorption factor of the *i*_th_ surface relative to the other *j* surfaces. *A*_*j*_ is the area of the *j*_th_ surface. *σ* is the Stefan Boltzmann constant 5.67 × 10^− 8^ *W/(m*^*2*^$\:\bullet\:{k}^{4}$*). k*_*ij*_ is the thermal combination parameter of the *i*th node with the relevant node *j*. *T*_*j*_ is the temperature of node *j*, and T_*i*_ is the temperature of node *i. ε*_*ii*_ and *ε*_*ei*_ on the right side of the equation represent the emissivity of the node *i* itself, including the radiation to the inner surface and to space, for the nodes on the imager surface. *m*_*i*_ is the mass of node *i*, *c*_*i*_ is the specific heat capacity of node *i*, and *τ* is time.

The first term on the left side of Eq. (2) represents the heat flux that is absorbed by the *i*_th_ surface, including heat from solar radiation, Earth reflection and Earth infrared radiation. The second term represents the proportion of the energy that is emitted by all surfaces of the spacecraft that is then absorbed by the *i*_th_ surface. The third item (*Q*_*i*_) represents the internal heat source. The fourth term represents the heat transfer of the *i*_th_ surface to the other surfaces via thermal connections. The first term on the right side of the equation represents the heat of the *i*_th_ surface radiated inside and outside the terminal. The second term indicates the change in the internal energy of the *i*_th_ surface.

#### Radiation heat transfer

The radiation heat transfer formula between spacecraft and space is as follows:

3 $$Q=\sigma \cdot \varepsilon \cdot A \cdot F(T_{1}^{4} - T_{0}^{4})$$

where *Q* represents power, σ is the Steffen-Boltzmann constant with a value of 5.67 × 10^− 8^ W/m^2^/K^4^, *ε* is the infrared hemisphere emissivity, *A* is the area of the radiating surface (unit: m^2^), *F* is the angle of view coefficient, *T*_*1*_ is the temperature of the radiating surface (unit: K); *T*_*0*_ is the temperature of the cold black background of the universe (unit: K), which can be ignored from the perspective of thermal design.

#### Thermal finite element model

The finite element model is used to evaluate the thermal design scheme by clearly defining the temperature distribution on the surface of the laser communication terminal^15^. During the optimization of the thermal design scheme, the model is frequently used for thermal simulation, so the calculation efficiency of the model is crucial. Time-consuming models slow down the optimization process, while fast but inaccurate models can lead to meaningless results.

**Fig. 7:**
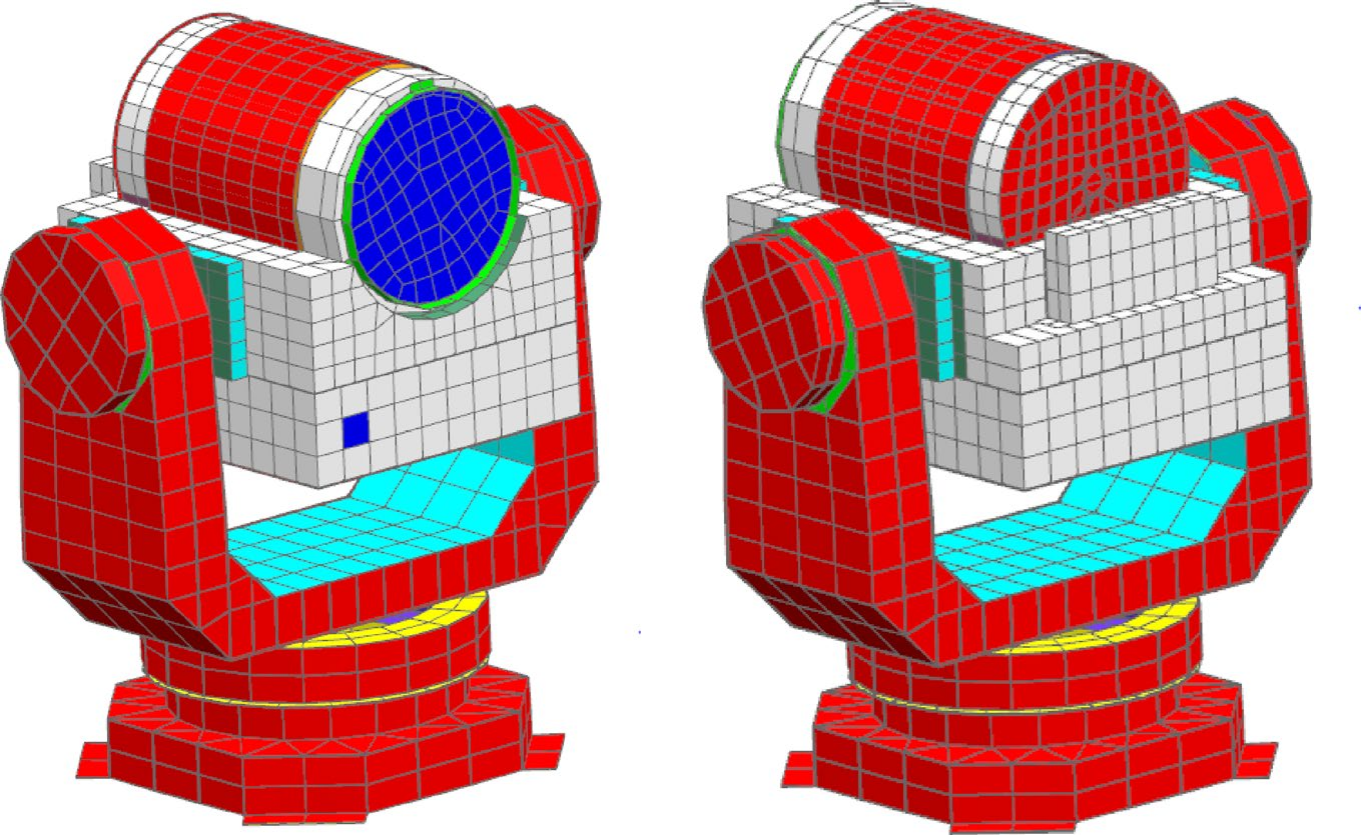
Thermal finite element model.

Considering the complexity of the structure, the cost of modeling and calculation, it needs to be simplified reasonably when modeling. On the premise that the total heat capacity of each component is unchanged, the small local features such as mounting holes and bosses in the parts are appropriately simplified. Ignore the influence of some small structural parts on the heat conduction and radiation shielding of the model, such as electrical connectors, cable cables, equipment installation screws, etc. Combined with the thermal control design scheme, the structure is simplified with the NX/SST software, and the thermal model is shown in Fig. 7. The model is manually divided into 5094 elements. In the thermal simulation of the manuscript, the calculation duration is 6 orbital periods, the time step is set to 30 s, and the convergence accuracy is 0.1℃.

### Analysis of external heat flux and determination of thermal conditions

The space heat fluxes mainly include three parts: solar radiation heat flux, Earth’s albedo heat flux and Earth’s infrared radiation heat flux. Among them, the Earth’s infrared radiation heat flux is only related to the orbit height and does not change much during its lifespan. Therefore, the changes in space heat fluxes are mainly reflected in solar radiation heat flux and Earth’s albedo heat flux, which are caused by the periodic motion of the sun in the ecliptic plane, resulting in the β-angle between the solar light vector and the satellite’s orbital plane changing within a certain range. Therefore, the changes in the comprehensive energy of orbit heat fluxes during satellite operation in-orbit depend on β-angle.

**Fig. 8:**
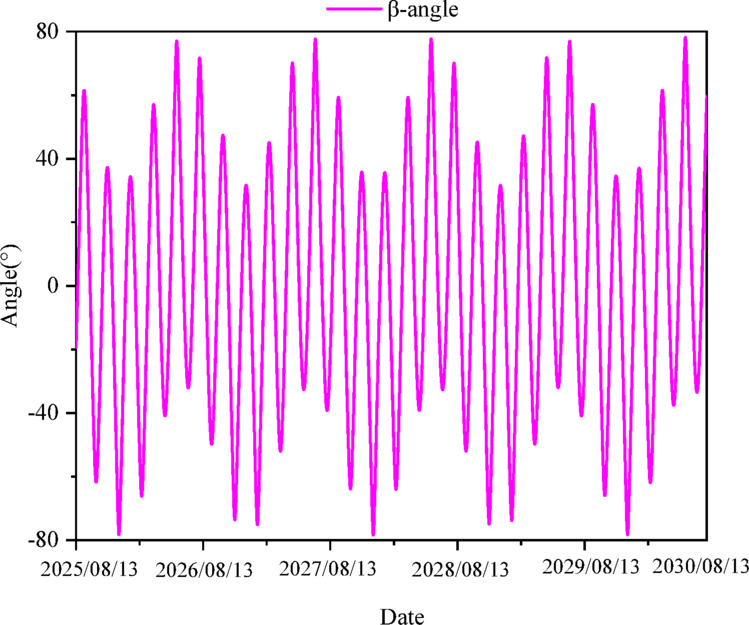
β-angle varying with time (5 years).

Figure 7 shows the variation of the satellite’s β-angle over the past 5 years. As can be seen from the Fig. 8, the satellite’s β-angle varies periodically within the range of -78.4° to 78.4°. When the absolute value of the β angle is greater than 68°, the orbit is in the full illumination period.

**Fig. 9:**
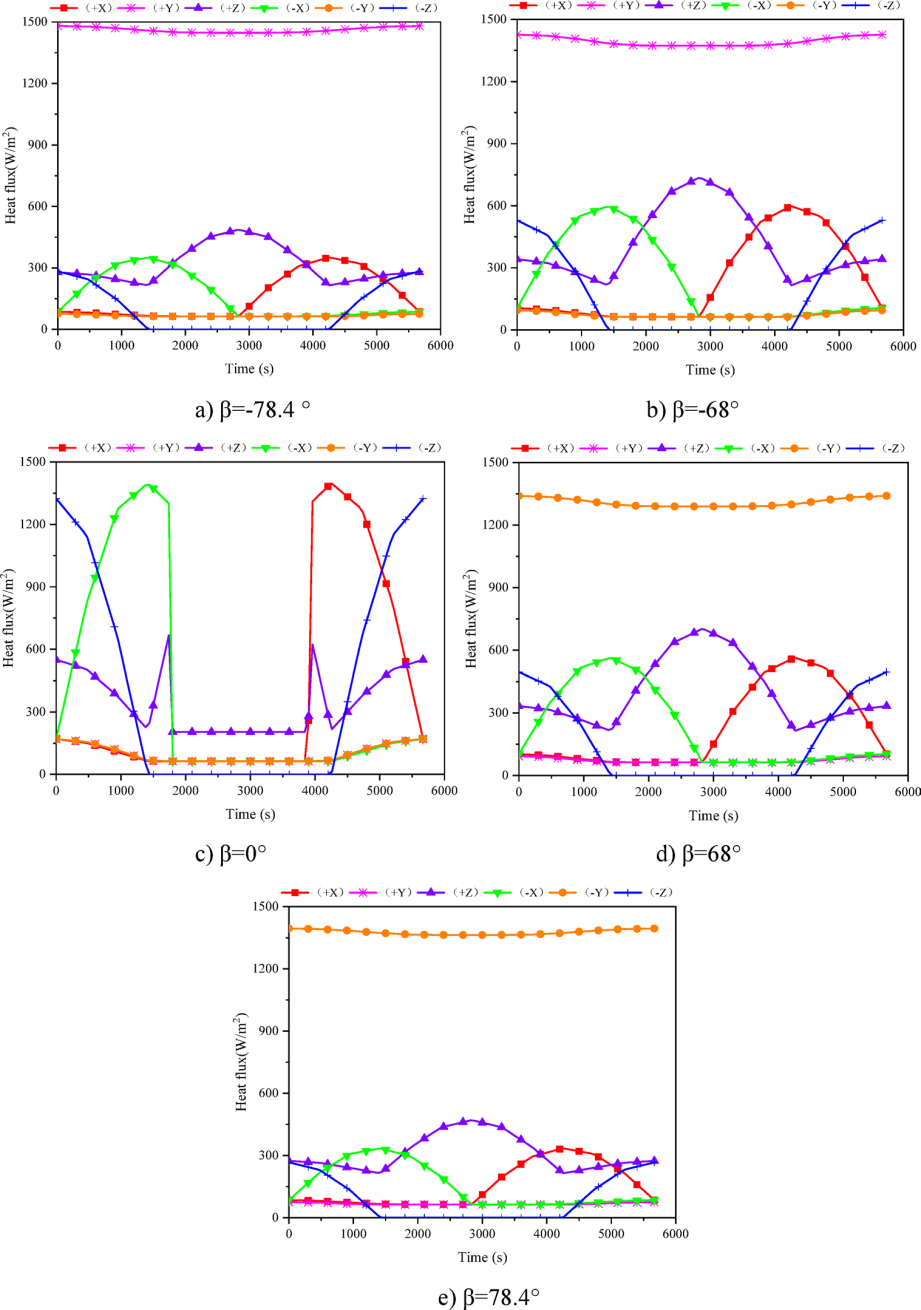
External heat flux curves of different surfaces of laser communication terminal (**a**) β=-78.4 ° (**b**) β=-68° (**c**) β = 0° (**d**) β = 68° (**e**) β = 78.4°.

To determine the position of the radiating surface and the extreme conditions, several specific β angles are selected to calculate the external heat flux, namely ± 78.4°, ± 68° and 0°. The calculated results of the external heat fluxes of different surfaces are shown in Fig. 9.

To comprehensively evaluate the external heat flux received by laser communication terminal in-orbit, the average external heat fluxes at different β angles on each surface are counted, as shown in Table 3. Since the -Y surface of the laser communication terminal is completely blocked by the satellite, the external heat flux of this surface is no longer taken into account.

**Table 3:** Average external heat flux arrived of laser communication terminal (W/m^2^).

β-angle	+X surface	+Y surface	+Z surface	-X surface	-Z surface	Total
– 78.4°	158.3	1461.4	316.9	158.3	91.1	2186.0
– 68°	238.3	1393.7	411.2	242.9	171.4	2457.4
0°	374.6	98.3	345.7	379.3	426 0.3	1624.2
68°	228.6	72.0	399.3	228.5	159.5	1088.0
78.4°	152.9	66.5	310.4	152.8	85.9	768.4

From the above figure and table, it can be seen that the + Y surface is exposed to sunlight for a long time when the β angle is negative, resulting in a large external heat flux. However, when the β angle is positive, it is shaded, and the external heat flux is smaller. The -Y surface changes are opposite to the + Y surface. The external heat flux received by the + Z and -Z surfaces is relatively stable. The external heat flux of the + X and -X surfaces is the largest when the β-angle is 0°, and the periodic changes are large.

Given the external heat flux values arrived the laser communication terminal, the external heat flux is at the highest level with the β-angle of -68°, which can be regarded as a hot-case. The external heat flux is at the lowest level with the β-angle of 78.4°, which can be regarded as a cold-case. The definition of the thermal analysis conditions is shown in Table 4. The main material parameters used in the thermal simulation are listed in Table 5.

**Table 4:** The definition of the thermal analysis conditions.

Number	Name	Parameter setting
1	Cold- case	The solar constant is 1322 W/m². The thermal control coating parameters are taken at early-life. Except for the heat source of the beacon component, all other internal heat sources are operating normally. The active heating circuit is working. The terminal is in a zero-attitude state.
2	Hot-case	The solar constant is 1414 W/m². The thermal control coating parameters are taken at end-life. All internal heat sources are operating normally. The active heating circuit is working. The terminal is in a zero-attitude state. The azimuth axis of the terminal rotates by 90°.

**Table 5:** Thermal physical parameters of the main materials.

Number	Name	Thermal conductivity (W/m/K)	Specific heat capacity (J/kg/K)	Emissivity	Solar absorptivity	
					Early	End
1	Aluminum-based silicon carbide (30%)	175	860	0.8	0.8	0.8
2	Silicon	148	700	0.8	0.8	0.8
3	Invar steel (4J32)	13.9	450	0.8	0.8	0.8
4	Titanium alloy (TC4)	5.4	611	0.08	0.52	0.52
5	Aluminum alloy (6061)	167	900	0.85	0.85	0.85
6	White paint	/	/	0.92	0.12	0.25
7	MLI (polyimide film)	0.05	400	0.67	0.36	0.6

### Thermal analysis results

**Fig. 10:**
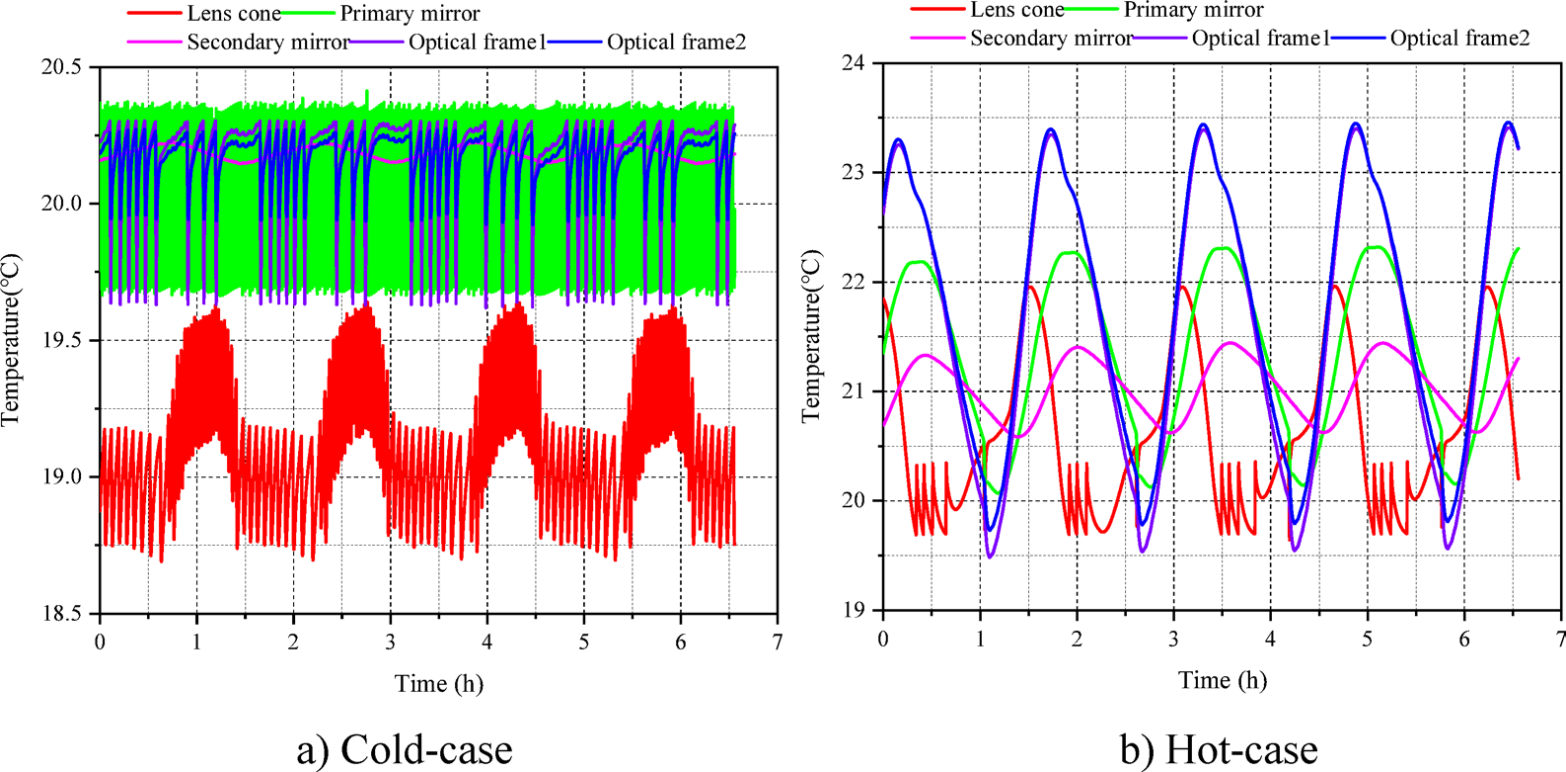
The temperature curve of the optical system (**a**) Cold-case (**b**) Hot-case.

According to the thermal design scheme and the planning of thermal analysis conditions, thermal simulation calculations for cold-case and hot-case are carried out, and the temperature curve of the optical system is shown in Fig. 10. The temperature data of key components are summarized based on the thermal simulation results, as shown in Table 6. From the temperature curve and data, it can be seen that the temperature of each component can be controlled near the target temperature and meets the temperature index requirements, indicating that the thermal design scheme is very effective.

**Table 6:** Thermal simulation temperature statistics (℃).

Number	Name	Cold-case	Hot-case	Temperature index
1	Lens cone	18.7 ~ 19.6	19.5 ~ 22.0	20 ± 3
2	Secondary mirror	20.1 ~ 20.2	20.6 ~ 21.4	20 ± 3
3	Primary mirror	19.7 ~ 20.4	20.1 ~ 22.3	20 ± 3
4	Optical frame1	19.6 ~ 20.3	19.5 ~ 23.4	20 ± 5
5	Optical frame2	19.9 ~ 20.3	19.8 ~ 23.5	20 ± 5
6	Beacon circuit box	10.3 ~ 11.2	23.0 ~ 39.0	-15 ~ 50
7	U-frame 1	14.7 ~ 15.3	16.4 ~ 17.7	5 ~ 35
8	U-frame 2	14.7 ~ 15.3	18.1 ~ 19.4	5 ~ 35
9	Base	14.7 ~ 15.3	23.4 ~ 24.3	5 ~ 35
10	Rotation shaft	14.8 ~ 15.2	24.9 ~ 25.8	5 ~ 35

## Thermal balance test verification

The thermal balance test is the most important means to verify the thermal control design effect of laser communication terminal. Through the thermal balance test, the correctness of the thermal design can be verified, and the temperature distribution data of the laser communication terminal can be obtained. In addition, by comparing and analyzing the results of the thermal balance test with those of the thermal analysis, deficiency can be identified^16^, such as the finite element model, material properties, working loads, initial conditions and boundary conditions. It can also provide guidance for thermal design optimization.

### Test scheme

**Fig. 11:**
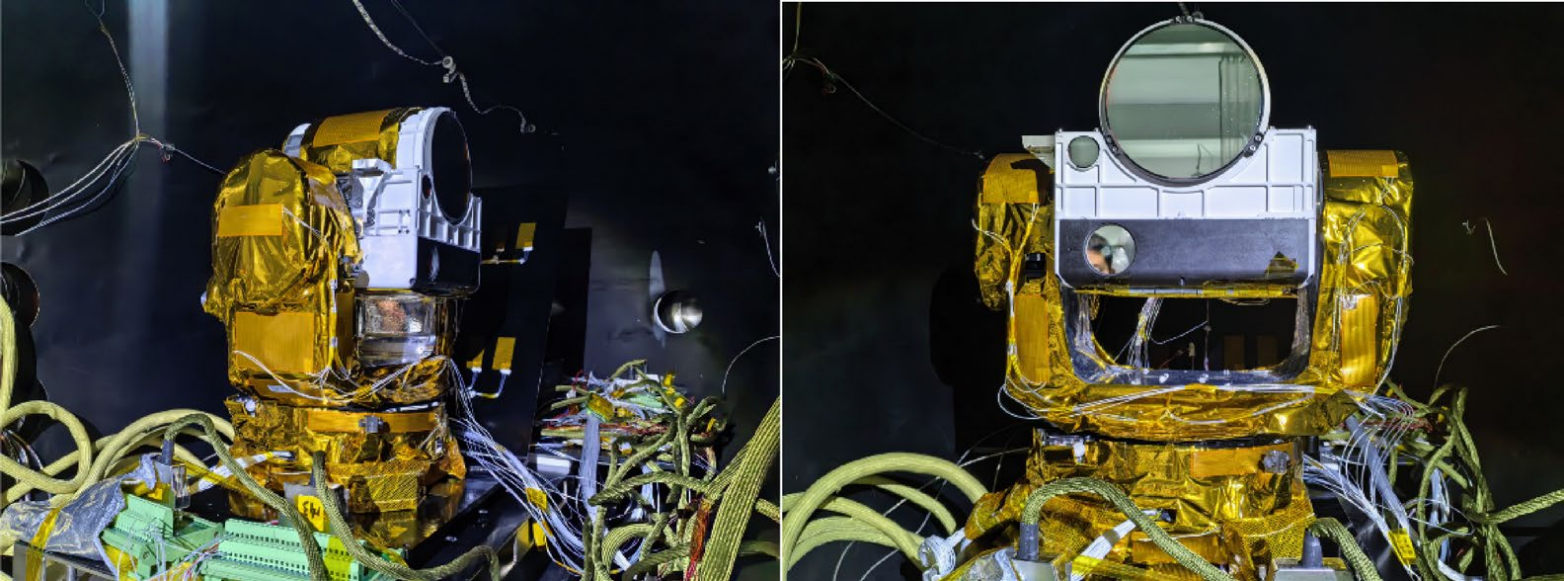
A layout diagram of the laser communication terminal in the vacuum tank.

Figure 11 shows the layout of the laser communication terminal in the vacuum tank. Liquid nitrogen is introduced into the vacuum tank to maintain the temperature at approximately 95 K, simulating the temperature of the space environment. The real-time temperature of the laser communication terminal in the vacuum tank is collected by the temperature measurement and control equipment, and the external heat flux in-orbit is simulated by the heater.

### Test results

**Fig. 12:**
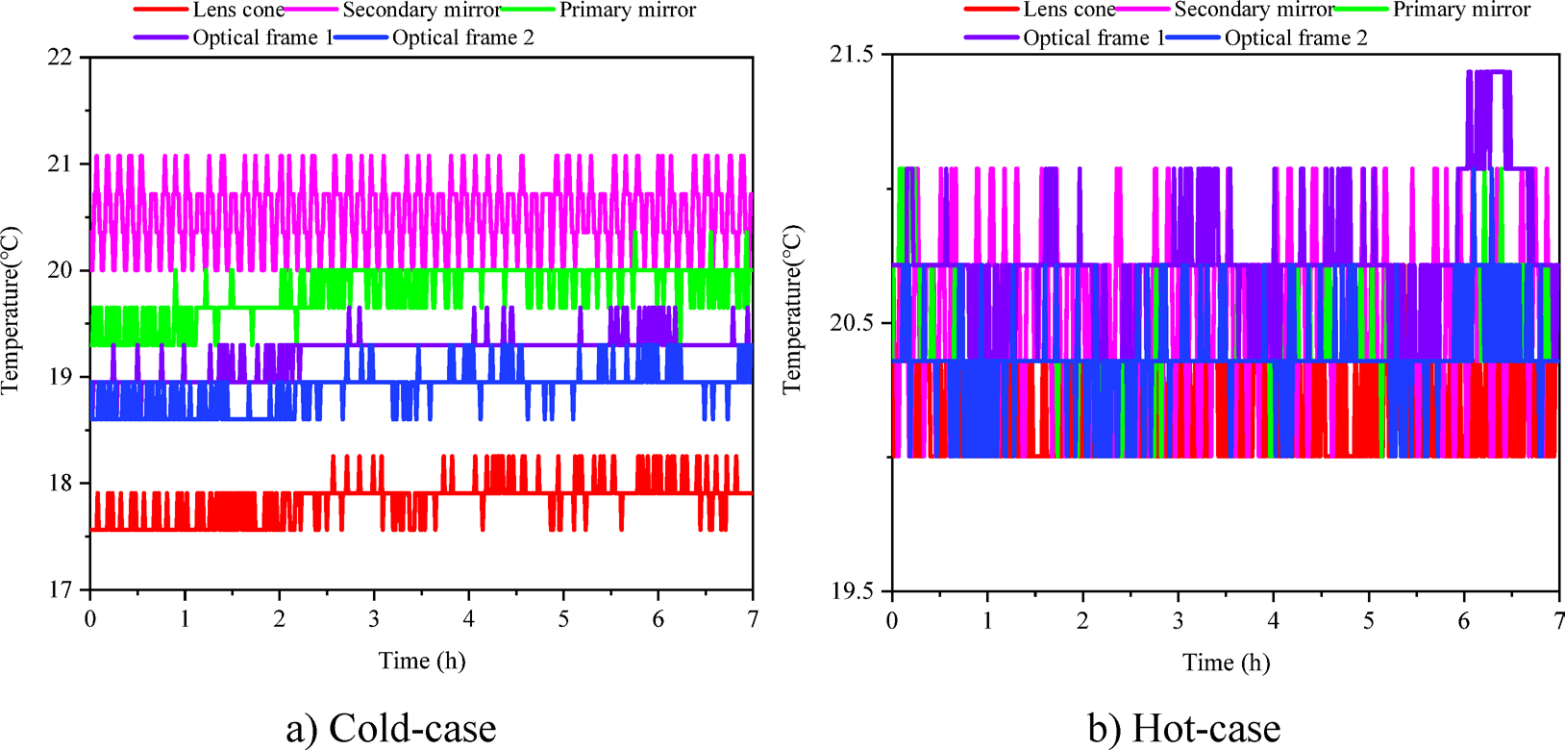
Temperature curve of optical system (**a**) Cold-case (**b**) Hot-case.

According to the defined hot-case and cold-case, thermal balance tests are conducted. The temperature curve of the optical system is shown in Fig. 12, and the temperature data for key components is presented in Table 7. From the chart, it can be seen that the temperature of the optical system can be controlled close to the target temperature, and the temperatures of all components meet the temperature index.

**Table 7:** Statistical data of temperature in thermal balance test (℃).

Number	Name	Cold-case	Hot-case	Temperature index
1	Lens cone	17.6 ~ 18.3	20.0 ~ 20.7	20 ± 3
2	Secondary mirror	20.0 ~ 21.1	20.0 ~ 21.1	20 ± 3
3	Primary mirror	19.3 ~ 20.4	20.0 ~ 21.1	20 ± 3
4	Optical frame1	19.3 ~ 19.7	20.4 ~ 21.4	20 ± 5
5	Optical frame2	18.6 ~ 19.3	20.0 ~ 21.1	20 ± 5
6	Beacon circuit box	9.6 ~ 10.6	10.2 ~ 32.9	-15 ~ 50
7	U-frame 1	11.9 ~ 12.5	15.2 ~ 15.9	5 ~ 35
8	U-frame 2	14.9 ~ 15.9	17.9 ~ 19.3	5 ~ 35
9	Base	12.2 ~ 12.9	21.8 ~ 23.3	5 ~ 35
10	Rotation shaft	8.9 ~ 9.3	23.6 ~ 24.4	5 ~ 35

**Fig. 13:**
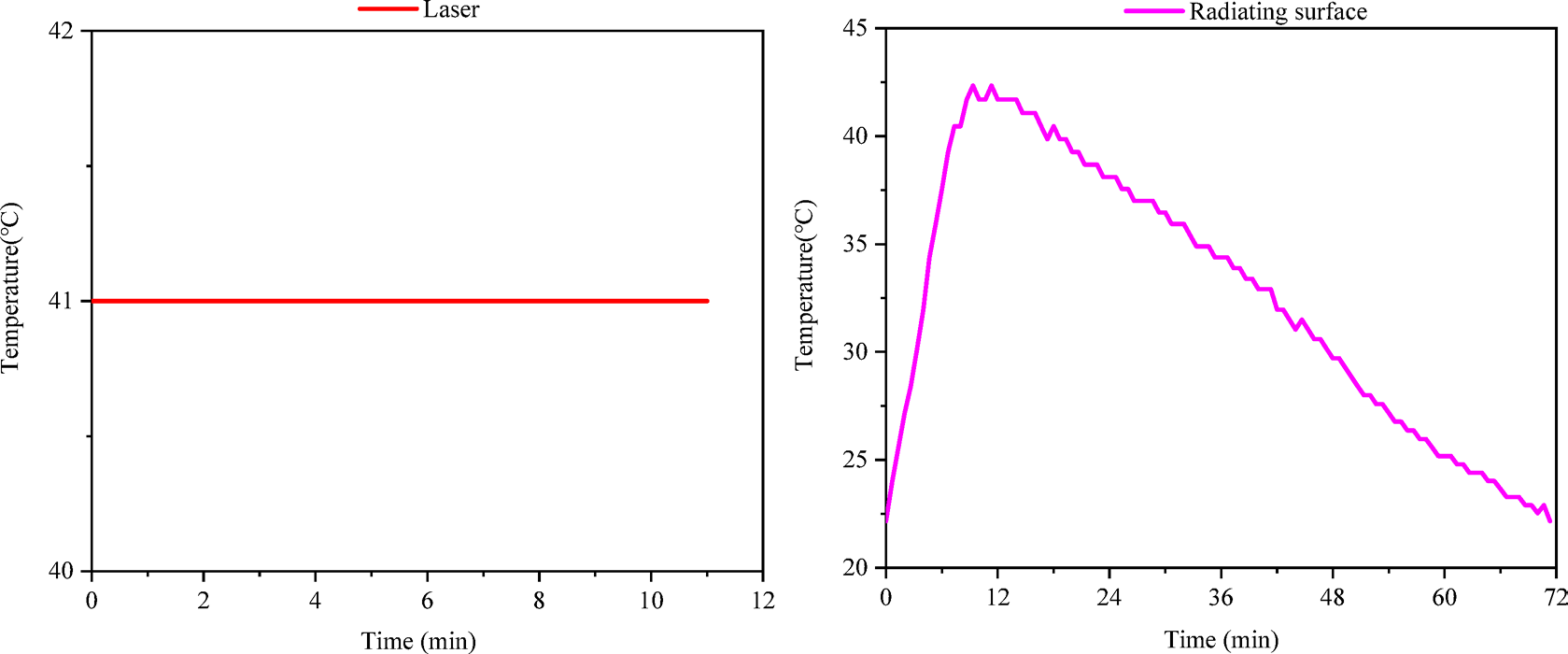
Temperature curves of beacon components.

Additionally, in hot-case, test is conducted on the beacon component, which lasts for 11 min. During the first 4 min, only the beacon circuit is powered on for working, while in the following 7 min, both the beacon circuit and the laser are powered on simultaneously. Throughout the entire process, the laser temperature is always maintained at 41 °C, and the temperature of the laser’s radiating surface rises from 22.2 °C to 42.3 °C. Subsequently, the beacon circuit and the beacon laser are powered off, and after 60 min, the temperature of the laser’s radiating surface decreased from 42.3 °C to 22.2 °C. From the test result of the beacon component, it can be seen that the beacon component can meet the usage requirements of working in-orbit for 5 min, and there is still a considerable margin. The temperature curves of the beacon laser and the radiating surface are shown in Fig. 13.

### Results analysis and discussion

From the results of thermal simulation and thermal tests, it can be seen that the temperature obtained from the thermal simulation is slightly higher than that from the thermal test. Among the components, the ones with larger temperature deviations are the shaft components (U-frame(2.8℃), rotation shaft(5.9℃), base(2.5℃)) and the beacon circuit (3.3℃), while the ones with smaller deviations are the main optical system (1.3℃), as shown in Table 8.

The results between thermal simulation and the thermal test cannot be 100% consistent. In general, the absolute value of the maximum error between them is no more than 5℃. In Table 8, the maximum error for the rotation shaft is 5.9℃, followed by the beacon circuit box with an error of 3.3℃, and the maximum error for the optical system is 1.3℃. Except for the rotation shaft, all the other components meet the error requirements. In fact, the reason for the large error of the rotation shaft is the insufficient heating power. The temperature control target for the rotation shaft is 15℃. During the thermal test, due to insufficient heating power, its lowest temperature is only 8.9℃, deviating from the target temperature(15℃) by 6.1℃. In the future, this issue can be addressed by increasing the heating power of the rotation shaft to meet the error requirements.

**Table 8:** Results comparison of thermal simulation and thermal test (℃).

Name	Thermal simulation	Thermal test	Maximum deviation
Lens cone	18.7 ~ 22.0	17.6 ~ 20.7	1.3
Secondary mirror	20.1 ~ 21.4	20.0 ~ 21.1	0.9
Primary mirror	19.7 ~ 22.3	19.3 ~ 21.1	1.2
Optical frame	19.5 ~ 23.5	18.6 ~ 21.4	2.1
Beacon circuit box	10.3 ~ 39.0	9.6 ~ 42.3	3.3
U-frame	14.7 ~ 19.4	11.9 ~ 19.3	2.8
Base	14.7 ~ 24.3	12.2 ~ 23.3	2.5
Rotation shaft	14.8 ~ 25.8	8.9 ~ 24.4	5.9

The reason for the large temperature deviation of the beacon circuit is that the working duration and heat consumption during thermal simulation and thermal test are inconsistent. Specifically, the working time of the beacon circuit during thermal simulation is 5 min, and the heat consumption is subject to constant value. During the thermal test, the working time is 11 min, and the actual heat consumption gradually increases. In the cold-case of the thermal test, the heating power of the shaft components is insufficient, causing the temperature of the shaft components to be lower than the temperature target. However, during thermal simulation, the temperature of the shaft components can all be controlled close to the target temperature.

During the thermal test, the low temperature of the laser communication terminal mainly has two reasons: (1) The active components cannot be fully covered by MLI, resulting in severe heat leakage. (2) To adapt to the large pitch and rotation of the laser communication terminal, a new wiring process is adopted to ensure that the cables would not interfere with rotation. However, the new wiring process also introduced new problems. After the heating circuit cables passed through the flexible belt, the resistance increased significantly, resulting in large power loss, as shown in Table 9. The power loss range of the heating circuit with the new wiring process is 16.0% ~38.8%, and the larger the designed power, the greater the loss. The heating circuit without the new wiring process has a power loss of only 2.5%.

To solve the problem that the temperature of the shaft components is low during the thermal test, the following measures can be taken: (1) Reducing the temperature target of the shaft components (for example, reducing from 15℃ to 10℃). (2) Appropriately increasing the heating power. Regarding the problem of large power loss in the new wiring process, it is recommended that the heating circuit is about 60% of the allowance during thermal simulation. In addition, when the heating power remains unchanged, a larger voltage can be selected to reduce power loss.

**Table 9:** Comparison of design values and actual values for heating power.

Name	Design value		Actual value		Power loss	New wiring process
	Resistance value(Ω)	Heater power(W)	Heating circuit resistance value(Ω)	Heater power(W)		
Lens cone	18	8	23	4.9	38.8%	Yes
Secondary mirror	36	4	40.9	3.1	22.5%	Yes
Primary mirror	28.8	5	33.8	3.6	28.0%	Yes
Optical frame1	12	12	15.2	7.5	37.5%	Yes
Optical frame2	12	12	15.2	7.5	37.5%	Yes
Beacon circuit box	24	6	29.1	4.1	31.7%	Yes
U-frame 1	28.8	5	31.6	4.2	16.0%	Yes
U-frame 2	28.8	5	31.5	4.2	16.0%	Yes
Base	36	4	36.3	3.9	2.5%	No
Rotation shaft	36	4	36.3	3.9	2.5%	No

## Conclusion

This paper discusses the main challenges faced in the thermal design process of the new generation of ultra-high-speed inter-satellite laser communication terminal. The influence of the satellite platform’s thermal interface is analyzed, and a detailed thermal design of the laser communication terminal is carried out with the thermal control difficulties. Subsequently, thermal simulation and thermal balance tests are conducted. The results of thermal simulation and thermal balance test show that the temperature of the laser communication terminal all meet the temperature index, and the thermal design scheme is reasonable and feasible. In addition, the reasons for the low temperature of the shaft components during the thermal test and the problems brought by the new wiring process are discussed, and solutions are provided, which provides a basis for the thermal design optimization of subsequent project batch production.

## Data Availability

All data generated or analysed during this study are included in this article.
